# NCS Assessments of the Motor, Sensory, and Physical Health Domains

**DOI:** 10.3389/fped.2021.622542

**Published:** 2021-11-26

**Authors:** Jane E. Clark, Russell Pate, Rose Marie Rine, Jennifer Christy, Pamela Dalton, Diane L. Damiano, Stephen Daniels, Jonathan M. Holmes, Peter T. Katzmarzyk, Susan Magasi, Ryan McCreery, Kerry McIver, Karl M. Newell, Terence Sanger, David Sugden, Elsie Taveras, Steven Hirschfeld

**Affiliations:** ^1^Department of Kinesiology, School of Public Health, University of Maryland, College Park, MD, United States; ^2^Department of Exercise Science, Arnold School of Public Health, University of South Carolina, Columbia, SC, United States; ^3^Specialty Therapy Source, Jacksonville, FL, United States; ^4^Department of Physical Therapy, University of Alabama, Birmingham, AL, United States; ^5^Monell Chemical Senses Center, Monell Center, Philadelphia, PA, United States; ^6^Rehabilitation Medicine Department, National Institutes of Health, Bethesda, MD, United States; ^7^Department of Pediatrics, University of Colorado School of Medicine, Denver, CO, United States; ^8^Department of Ophthalmology and Vision Science, University Arizona, Tucson, AZ, United States; ^9^Pennington Biomedical Research Center, Louisiana State University, Baton Rouge, LA, United States; ^10^Department of Occupational Therapy, University of Illinois at Chicago, Chicago, IL, United States; ^11^Boys Town National Research Hospital, Boys Town, NE, United States; ^12^Arnold School of Public Health, University of South Carolina, Columbia, SC, United States; ^13^Department of Kinesiology, University of Georgia, Athens, GA, United States; ^14^Department of Biomedical Engineering, Neurology, and Biokinesiology, University of Southern California, Los Angeles, CA, United States; ^15^School of Education, University of Leeds, Leeds, United Kingdom; ^16^Department of Pediatrics, Harvard Medical School and Mass General Hospital for Children, Boston, MA, United States; ^17^National Institutes of Health, Bethesda, MD, United States

**Keywords:** motor, sensory, physical health, children, assessment, National Children's Study

## Abstract

As part of the National Children's Study (NCS) comprehensive and longitudinal assessment of the health status of the whole child, scientific teams were convened to recommend assessment measures for the NCS. This manuscript documents the work of three scientific teams who focused on the motor, sensory, or the physical health aspects of this assessment. Each domain team offered a value proposition for the importance of their domain to the health outcomes of the developing infant and child. Constructs within each domain were identified and measures of these constructs proposed. Where available extant assessments were identified. Those constructs that were in need of revised or new assessment instruments were identified and described. Recommendations also were made for the age when the assessments should take place.

## The Motor Domain^1^

Daniel Wolpert in a TED talk recently offered the surprising premise that the brain evolved to afford animals with the ability to move, not to think or feel (1). Whether one accepts Wolpert's premise or not, it is clear that the human brain is challenged to control and coordinate its multi- segmented body in the service of achieving desired goals. Born unable to manage this complex, unwieldy body, a year after birth (on average), the typically developing human infant is able to rise, stand, and walk independently and self-feed with the early forms of communication (speech and gesturing) in place.

From the perspective of the motor domain committee, these motor capabilities may be broadly conceptualized as the control and coordination of the following functions: mobility, manipulation, posture, and communication. Mobility denotes the human's capability to move the body from one location to another (from sitting up to crossing the room). Manipulation refers to the ability to interact with the environment. Posture underlies mobility and manipulation, as it is the ability to assume desired positions and to stably maintain these positions. The human's motor capability to communicate takes several forms: speaking (vocalization), gesturing, writing, and keyboarding.

### Motor Domain Value Proposition: Importance of Motor Function Assessment for Health Outcomes

Movement is a signature feature of all living systems. Indeed, the human infant produces spontaneous and reflexive movements at birth, but it is the later emerging goal-directed intentional actions that are critical to the infant's survival and healthy well-being. Feeding, self-care, ambulation, and communication are critical motor functions for the infant to master. The vast majority of developmental screening tools include assessment of motor function (2). Owing to the predicable motor sequences of function (i.e., rolling over, sitting, standing, and walking, reaching, and self-feeding), clinicians have long used these behaviors to signal infants whose development is delayed or off course. For example, see the statement of the American Academy of Pediatrics Neuromotor Screening Expert Panel (3) and websites designed for parents and caregivers that also include these motor function milestones (4, 5).

Motor impairments (often appearing very early in development) are pervasive across many disorders. For some disorders, the motor difficulty is the primary defining feature; for example, cerebral palsy and Developmental Coordination Disorder (DCD). For other disorders, the motor difficulty is a co-occurring feature; for example, autism, ADHD, specific language impairment (SLI), and dyslexia. For both groups of children, their motor difficulties restrict their life choices, including restricting their participation in physical activity and thereby potentially impacting their health outcomes.

Later, motor skill competence provides the foundation for a lifetime of physical activity in culturally promoted games, dance, and sports. In the United States, the two leading causes of death are heart disease and cancer. Both the World Health Organization (WHO) and the Center for Disease Control (CDC) have identified physical inactivity as a major risk factor for death owing in large part to links to cancer, diabetes, and cardiovascular disease (6, 7). Evidence is now accumulating that the motor skill competence acquired in early childhood is a mediator of later physical activity and fitness [e.g., (8–12)].

Additional evidence is accumulating on the importance of physical activity on cognitive function. This evidence first emerged for older individuals, but a considerable literature is developing showing the positive effects of physical activity on children's cognitive function (13–15). Indeed, as early as Piaget (16), psychologists have recognized the importance of motor actions on cognition. Today, the concept of “embodied cognition” affirms the importance of the sensorimotor origins of thinking (17, 18). Recently, the Avon Longitudinal Study of Parents and Children (ALSPAC), an on-going population-based study in the United Kingdom, recently reported that moderately vigorous physical activity may be beneficial for attentional processes in adolescents, particularly males (19).

Motor function competence also may be a mediator in social-emotional health. Research has shown that motor impairments in both pre-school and school-age children impact emotional health (20, 21). For example, children with DCD were found to withdraw and avoid motor activities (22), have poorer self-perceptions and self-worth (23, 24) and are more likely to have higher levels of anxiety (24, 25) and depression (26).

### Motor Domain Constructs

Motor function has many dimensions that are related to health outcomes. Three constructs are particularly important, and a fourth should be considered for inclusion. The three essential constructs for assessment include: mobility, manipulation, and posture. The fourth, communication, may overlap with constructs in the cognitive domain.

Clearly the assessment of the “motor domain function” is not the same as the assessment of the “physical domain” where physical assessments are made of the body's anthropometrics (e.g., height, weight, etc.), physiological functioning (e.g., lung capacity, blood pressure, and heart rate) and so on. Certainly, these assessments may mediate motor function, but they should not be construed as representing the assessment of the motor “functioning” that provides the neuromotor control and coordination of purposeful actions.

#### Motor Domain Construct: Mobility

The human body is comprised of connected segments (e.g., trunk, head, upper, and lower limbs). Mobility is the ability to move the body from one place to another. This ability requires the control and coordination of the multi-segmented body to achieve a desired orientation and stability. Once the infant is able to walk independently, mobility has been measured by the speed of bipedal locomotion (e.g., NIH Toolbox). However, this is a very limited measure as it involves only upright bipedal mobility and assesses only one dimension (i.e., speed). Indeed, the literature includes a variety of measures assessing many dimensions including: rising from a seated position changing levels, gait initiation, agility (changing directions quickly), various locomotor forms (i.e., various interlimb coordination such as hopping, skipping, and galloping), and the forceful projection of the body upward (vertical jump) or forward (standing long jump). For the NCS we recommend using three sub-constructs of mobility: (1) general mobility; (2) locomotor skills; and, (3) forceful body projection.

#### Motor Domain Construct: Manipulation

The ability to act with and upon objects in the environment is an essential skill for the developing infant and child. Self-feeding, tool-use, dressing, handwriting and use of standard information technologies (computer, tablet, smart phone) and gestures are all considered part of the construct of “manipulation.” Manipulation is a multi-dimensional construct that requires motor planning (praxis), perceptual-motor integration, upper-limb segmental control, and finger dexterity. Hand function is a complex multi-dimensional construct that requires the coordination and integration of different parts of the cerebral cortex to enable the precise and smooth control of hand movements. The capacity for sophisticated hand-object interaction is a characteristic of higher primates and critical to our interactions with the world. Hand-object interaction (or manipulation) requires independent finger movement, somatosensory guidance of movement, and the ability to transform sensory information about object properties into appropriate movement patterns and hand configurations. Motor control of the intrinsic muscles in the hand provides the child with the ability to handle small objects with precision. The brain must constantly adapt to changing environmental circumstances to allow for precise and efficient hand use. The primary motor cortex not only controls movement but it is part of a dynamic system and is thus changed as a result of use through a process of motor learning.

In the period from birth to ~6 ½ years, children develop new movement patterns and progressively disassociating their hand movements. For example, at 6 months, children can use a full fisted grasp (palmar grasp) to hold an object but over time the grasp is refined and proceeds through a known developmental sequence culminating for 50–70% of children in a dynamic tripod grasp. Thereafter, the developmental focus is on the increased refinement, efficiency and precision of hand functions. For example, while a child may be able to hold a pencil in kindergarten their writing is typically large, boxy, and slow. Over time, handwriting is refined and speed and accuracy increase. Effective performance of age-appropriate functional activities requires increasingly sophisticated integration of unilateral and bilateral hand function. Children must be able to effectively plan, coordinate and execute purposeful tasks with real world objects. Both speed and accuracy must be balanced for successful performance of manipulation-based activities. Assessment of children's abilities to plan and smoothly execute fine motor tasks with real and on- screen objects is critical to understanding how children interact with their physical world. Given the important relationship between handwriting proficiency and academic achievement, assessment of pre-writing through cursive is critical in the comprehensive assessment of children's development.

Four sub-constructs of manipulation are included for assessment: (1) prehension; (2) dexterity; (3) fine motor precision and accuracy; and (4) handwriting.

#### Motor Domain Construct: Posture

Moving the end of a limb (for reaching or stepping) or remaining in the same position requires a “backdrop” of postural control. The multi-segmented body is “deformable.” Every joint (at the neck, shoulder, elbow, wrist, hip, knee, ankle, and so forth) needs to be controlled so that the position of the segment on either side (e.g., the knee with the thigh and lower leg) is oriented to the environment and to other joints. There are two components of posture. The first involves the maintenance of a position relative to the environment and/or to another body part. The second involves changing positions to achieve a particular posture or orientation. One of the first motor milestones, head control, reflects controlling the head in a desired position and then maintaining that position. Throughout the 1st year of life, the infant's ability to change body positions or to maintain stability whilst standing and then walking demonstrates clearly the motor control and coordination challenge in assuming and maintaining posture.

Similarly, to reach for an object or to manipulate objects requires the same postural control to assume a particular posture to the environment as well with other body parts and to stabilize the limb as it manipulates or interacts with objects in the environment.

Beyond the 1st year of life, changes in body positions while maintaining overall stability in walking, running, throwing, catching and all the fundamental motor skills are reliant upon adequate postural control and stability. In children with motor difficulties, improving postural control has wide-ranging effects on their motor skills. Posture control is an essential component of nearly all motor activities and improving this function has the effect of supporting the child in activities of daily living.

There are two sub-constructs of posture included for assessment: (1) static balance; and (2) dynamic balance.

#### Motor Domain Construct: Communication

This motor function construct refers to the ability of the body to communicate with others. Obviously, vocalization (speech motor control) is the primary motor capability of the communication construct. But the communication construct in the motor function domain also includes non-verbal communication such as vocalizations (not speech), gesturing, handwriting, and keyboarding. The latter motor functions are often considered part of the manipulation construct. Similarly, speech and vocalization might also be relevant to cognitive and social-emotional correlates of health than directly to health outcomes. Thus, the communication construct is not recommended for inclusion in the motor domain assessment except for infant and toddler assessment from 3 to 24 m.

### Motor Domain Assessments Recommended

The following assessment instruments were recommended for the motor domain. Following the assessment's name are the ages recommended for the assessment as well as the specific motor construct(s) the instrument is designed to assess. The link for the NIH Toolbox for the motor domain assessment is: https://www.healthmeasures.net/explore-measurement-systems/nih-toolbox/intro-to-nih-toolbox/motor

**Infant & Toddler Developmental Assessment of Motor Milestones (IT- DAMM)** (Ages: 3–24 m). To assess: mobility, (general, locomotion) manipulation (prehension, dexterity), posture (static, dynamic), and communication (non-verbal, speech)
**Supine Timed Up and Go (S-TUG)** (Ages: 3 years onward). To assess: mobility (general, locomotion), posture (dynamic)
**Vertical Jump** (Ages: 5 years onwards). To assess: mobility (forceful body projection).
**NIH Toolbox 9-hole Peg Test** (Ages: 3 years onward). To assess: manipulation (prehension, dexterity
**Developmental Assessment of Manipulation (DAM)** (Ages: 4 years onward). To assess: manipulation (prehension, dexterity, fine motor precision/accuracy, handwriting).
**One-Leg Standing Balance Test** (3–7 years). To assess: posture (static).
**Walking Line Test of Dynamic Balance** (4 years onward). To assess: posture (dynamic).
**NIH Toolbox Balance Test** (ages 8–9 years onward). To assess: posture (static)

### Motor Assessment Schedule

In Table 1, the recommended motor assessments are listed along with the age recommended for their assessments. These ages were selected based on the ages of assessment to be included in the National Children's Study.

**Table 1:** Recommended motor assessments and age for assessment.

**Domain**	**Construct**	**Measure**	**Birth**	**8 m**	**14 m**	**21 m**	**3 y**	**4 y**	**5 y**	**7 y**	**9 y**	**11 y**	**13 y**	**15 y**	**17 y**	**19 y**	**21 y**
Motor	Milestones battery	Infant and Toddler Developmental Assessment of Motor Milestones^*^		X	X	X	X										
Motor	Mobility, posture	Supine Timed Up and Go (S-TUG)^*^					X	X		X	X	X					X
Motor	Mobility	Vertical Jump^*^						X		X	X	X					
Motor	Manipulation	NIH Toolbox 9-hole Peg Test					X		X	X							
Motor	Manipulation	Developmental Assessment of Manipulation (IT-DAMM)^*^						X	X	X							
Motor	Posture	One-Legged Standing Balance Test									X		X			X	
Motor	Posture	Walking the Line		X	X	X	X	X	X	X	X	X	X	X	X	X	X
Motor	Posture	NIH Toolbox Balance Test		X	X	X	X	X	X	X	X	X	X	X	X	X	X

* *To be developed*.

### Motor Assessments in Need of Development

Not all the motor domain assessment instruments were fully developed and standardized psychometrically. Therefore, the NCS Motor Domain Team proposed that the following instruments were still in need of further psychometric work.

#### Need Minimal Psychometric Work

Three of the assessment instruments needed minimal psychometric work. These included: (1) Vertical Jump (mobility construct); (2) One-leg standing balance (posture-static construct); and, (3) Walking a line (posture-dynamic construct).

#### Need More Substantial Psychometric Work

Three of the assessments instruments needed more substantial psychometric work. These included: (1) Infant and Toddler Assessment of Motor Milestones (ITAMM) (mobility, manipulation, posture, and communication for ages 3–24 m); (2) Supine Timed Up and Go (S-TUG) (mobility and posture construct); and, (3) Automated Developmental Assessment of Motor Function (ADAMF; manipulation construct).

## Sensory Domain^2^

Sensation refers to the ability to receive information from the body and one's environment *via* receptors of multiple modalities. These modalities include vision, hearing, the chemical senses (e.g., taste and olfaction), somatosensation and vestibular (movement and orientation in space). Multi-sensory processing and interpretation (e.g., perception) are critical to development, health and safety. Multisensory perceptions are dependent upon intact peripheral and central sensory systems, experience, environment, and behavior. The perceptions, in turn, affect development of cognition, emotion and motor abilities (27–29). Unlike most measures, measures of sensation and perception provide direct and indirect measures of the integrity of peripheral and central nervous system function and health. Therefore, measures of the various sensory modalities can provide a measure of the health of the nervous system. Additionally, measures of sensation are critical for valid interpretation of many behavioral measures. For example, problems with fine motor tasks may be caused by the inability to see (assessed by visual acuity, in the context of refractive error), decreased depth perception (assessed by stereoacuity and alignment), visual processing (assessed by reading and cognition) or diminished somatosensory processing (assessed by stereognosis and two-point discrimination) (30, 31). Development of balance and gross motor abilities can be impaired by peripheral vestibular or somatosensory deficits (32–35) or central sensory processing (36). Many environmental exposures (i.e., diet, infection or disease, injury, chemical) can disrupt sensory function. Consequently, measures of sensation are an important component of measurement of children's or developmental health as defined for the National Children's Study (NCS).

### Sensory Domain Value Proposition: Importance of the Sensory Function Assessment for Health Outcomes

As noted above, sensation is critical for typical development and function. A brief discussion of the specific importance of each of the sensory modalities and sub-constructs is provided below.

#### Chemical Senses

Recent research supports that functional measures of taste and olfaction provide markers for not only eating behavior pathologies (e.g., obesity, anorexia, and failure to thrive), but also for neuropsychiatric disorders such as autism and attention deficit disorder in childhood (37). Our taste system's key function in guiding food selection and intake makes it important to health and the prevention of chronic diseases and conditions (38). Therefore, evaluating constructs such as sensitivity and preference to different taste qualities at early ages can identify the potential for unhealthy food choices throughout life (39).

Our sense of smell provides information about our air, water, and food that is critical to health and safety, nutrition, and psychological well-being (40). There is substantial evidence that olfactory function, particularly odor identification ability, can serve as an early biomarker in disorders with dopaminergic pathology (e.g., attention deficit/hyperactivity disorder, autism, and schizophrenia), among children and adolescents (37). In addition, sports-related head injuries, particularly those that damage the orbito-frontal cortex, are a rising public health concern. This type of injury is frequently accompanied by deficits in olfactory function (41–43), and there is evidence that this damage is related to less favorable post-concussion outcomes (44, 45).

#### Hearing

Hearing allows humans to develop spoken language, which in turn impacts other areas of development such as cognition. Because peripheral inputs to the central auditory system shape the auditory cortex, the integrity of input is highly important in the first few years of life. What is lost cannot be re-captured later in life. Scientists have identified more than one critical period related to audition, and consequent spoken language development. Research results support that auditory deprivation due to congenital hearing loss results in atypical activity of the auditory cortex (46). Reportedly, children who have hearing loss but have it restored *via* cochlear implants by 1 year of age have superior spoken language development compared to children implanted at 2 or 3 years of age (47).

Transient hearing loss due to ear infections in childhood may also change patterns of auditory development. The results of investigations indicate that transient hearing loss may cause binaural hearing deficits lasting years after peripheral hearing returns to normal (48, 49). Recent work using animal models substantiates that auditory development is altered when monaural transient hearing loss is introduced and then removed, though underlying principles have not been studied (50). This problem is important since conductive hearing loss occurs with middle ear effusion, which is the most commonly diagnosed illness among children in the United States (51, 52). Children with recurrent otitis media are known to demonstrate deficits related to speech understanding in noise, known to be related to academic difficulty and social withdrawal.

#### Somatosensation

Somatosensory perception is multifaceted, depending on internal and external states. It contributes to cognitive processes (53), and research supports the idea that haptic sensations influence social judgments and decisions (54). The negative implication of diminished somatosensory function development is well-documented (55). It is important to note that peripheral sensory neuropathy causing mechanical and thermal hypoesthesia reportedly occurs in ~50% of children with Type I diabetes, but it is under-reported and rarely tested (30). Measures are critical not only for the identification of deficits in somatosensation, but for the prediction of neurodevelopmental status at school age (27, 29, 56).

Measures of somatosensory processing have been shown to identify children at risk for periventricular leukomalacia and other neurodegenerative disorders (27). Measures obtained on newborns has been shown to have excellent prognostic value for motor and cognitive performance at 5 years (56, 57). Additionally, somatosensation is a critical contributor to balance ability (58, 59), and thus measurement is critical for appropriate interpretation of balance tests.

#### Vestibular

The integrity of the vestibular system is relevant for typical motor and postural control development (32–34, 60–62), cognition and learning (62–67). The VOR is primarily responsible for gaze stabilization during most activities of daily living, which is critical for general function and reading ability (68–71). Measures of VSR have been shown to correlate with motor development and the acquisition of walking (33, 72). Therefore, VOR and VSR components of vestibular function are important for normal development and well-being in children (73). Increasing reports of both peripheral and central vestibular dysfunction in children (estimated to occur in ~30% of children <6 years of age), and the adverse effect on vestibular function by chronic otitis media, medications (e.g., ototoxicity by aminoglycosides and platinum chemotherapy) (74, 75), and mild traumatic brain injury (mTBI) or concussion, emphasize the importance of testing vestibular function throughout childhood and adolescence (35, 73, 76). Common diagnoses affecting the peripheral vestibular system in children include benign paroxysmal positional vertigo (77), vestibular neuronitis (78, 79), otitis media with effusion (80, 81), and drug-induced vestibulopathy (74, 75, 82, 83). Common central vestibular diagnoses include Benign Paroxysmal Vertigo of Childhood (36, 84, 85), vestibular migraine (86, 87), and mTBI (i.e., concussion), which is common in children who play sports such as soccer and football (88–91). The impact that peripheral and central vestibular dysfunction has on vision, learning, and balance warrants measurement of vestibular function from infancy through adolescence.

#### Vision

Visual impairment has a profound effect on other aspects of development (i.e., motor ability and cognition), and has been estimated to occur in 4% of children (92). It is therefore important to assess vision as a critical component of childhood development and how children currently interact with the world.

Visual impairment can be defined as a deficit in visual acuity, the smallest target that can be resolved. The most common cause of a visual acuity deficit in children is refractive error, resulting in optical blur and primarily treated with spectacles (92). Refractive error is also associated with the majority of cases of amblyopia, which is a central loss of vision due to prolonged blur and/or misalignment (93). Ocular misalignment (strabismus) is one of the most common eye conditions affecting children, and a common cause of amblyopia, which in turn is one of the most common causes of childhood visual impairment (94). The perception of depth (stereoacuity) develops rapidly in early childhood and is considered important for other aspects of development (95). Stereoacuity is severely degraded by strabismus (96), and is often degraded by untreated refractive error (97). Complex visual tasks include reading, involving higher-level visual processing, and may be impaired even when visual acuity is normal. Therefore, assessment of reading should be performed in addition to other vision constructs.

### Sensory Domain and Sub-domain Constructs

The sensory modalities or sub-domains included for the NCS project are: hearing, olfaction and taste, somatosensation, vestibular, and vision. Each of the sensory sub-domains or sensory modalities is discussed below (presented in alphabetical order), to include: definition, importance, constructs, description of tests recommended, and tests developed.

For each of the sensory modalities involved, the primary constructs include detection, avoidance, threshold and processing. For inclusion in the National Children's Study, the Sensory Team identified constructs within each sensory sub-domain and measures for each that: (1) are critical to typical development and health, (2) provide information critical to the interpretation of other behavioral measures (e.g., balance, locomotion, reading), and (3) are known to be affected by pathology, injury, or exposures during childhood (i.e., chronic otitis media is the most common reason that young children are seen by a medical professional, and this is known to have a negative effect on hearing acuity and vestibular function). Each of the modalities is briefly described here.

#### Chemical Senses

The human taste system consists of the perception of oral sensations that are usually described as having one or more basic qualities: sweet, salty, sour, bitter, or *umami*. These taste qualities facilitate consumption of foods high in nutrients and contribute to rejection of toxins (bitter). The function of the taste system involves both the responsiveness of the system to taste stimuli as well as the hedonic value of the sensation. The human olfactory system allows us to detect odors, recognize and discriminate odor qualities, and identify the sources of odors in our world. The perception of food flavor involves a combination of olfactory activation caused by odorous compounds released into the naso-pharynx retronasally through chewing, drinking, and deglutition, and the blending of taste (salty, sour, bitter, sweet, and umami) and oral somatosensory sensations (texture, heat, cold). The primary sub-constructs of the chemical senses are Taste and Olfaction. For Taste, the team identified four sub-constructs to measure: taste sensitivity, overall taste preference, sucrose preference, and regional taste sensitivity. For Olfaction, the team identified four sub-constructs: odor detection, odor identification, odor preference and hedonic response and flavor *via* retronasal olfactory detection.

#### Hearing

Hearing is the ability to detect and process acoustic stimuli. The human ear can detect sound across a wide range of frequencies between 20 and 20,000 Hz, though human speech only occurs between a narrow range of 250 and 8,000 Hz, and the dynamic range of hearing is around 120 dB. The ability to hear develops in tandem with development of the peripheral and central auditory systems. Auditory development occurs rapidly during the first few years of life and continues to early adolescence (98). Peripheral structures, including the canal, middle ear, and cochlea, are completely formed at birth. The cochlea, the sensory organ of hearing, is adult size by 20 weeks gestation but does not have adult-like acuity until 12 months of age (99). However, the auditory cortex is not adult-like until the teen years. The sub-constructs for hearing are hearing sensitivity (tympanometry and threshold) and speech understanding in noise.

#### Somatosensation

The somatosensory system involves peripheral receptors within the skin, muscles, bones, joints, and internal organs. Reception and central processing centers enable the sensory modalities of touch, temperature (thermoreceptors), proprioception (mechanoreceptors), and nociception (chemoreceptors and nociceptors). Consequently, this system enables detection and interpretation of environmental and internal stimuli necessary for typical development, balance ability and safety. Because it guides exploration of the environment by the infant/young child, an intact somatosensory system is critical to typical development of cognition and motor abilities, and provides information to prevent injury (e.g., avoidance of hot objects). Somatosensory development begins early in embryogenesis and continues to mature during the first 2 years of life, with myelination, synaptic development and increasing nerve conduction velocity (27). Typical development is dependent upon experience and use. The integrity and development of the system may be disrupted by numerous pathologies or injury (i.e., diabetes, major surgery, and severe burns) (30, 100). The sub-constructs recommended by the team include: detection of light touch and joint position sense, avoidance, vibratory threshold, and processing (stereognosis).

#### Vestibular

The peripheral vestibular system is comprised of three semicircular canals and two otolith organs that lie next to the cochlea in each inner ear, with receptors that respond to angular and linear accelerations of the head (head movements) and gravity. The vestibular system enables clear vision during head movement (68, 69), and postural control for balance (60, 61, 72). This happens through two sub-constructs: (1) the Vestibulo-Ocular Reflex (VOR) which enables clear vision during head movement, and (2) the Vestibulo-Spinal Reaction (VSR), which works with other descending motor tracts to enable balance and control of posture (73, 101). The vestibular system is fully developed by 20-weeks gestation (102). The VOR and VSR are present at birth, though not adult-like. The VOR rapidly matures over the 1st year of life (69, 103, 104). The VSR responses continue to develop throughout childhood and adolescence (73, 104, 105).

#### Vision

Vision can be broadly defined as a combination of the ability to see, to use the eyes together and to process visual information. Visual ability includes the sub-constructs of visual acuity, alignment, refractive error, stereoacuity, and visual processing. Visual acuity may be defined as the size of the smallest target that can be resolved. Refractive error can be defined as the optical deficit of the eye that needs to be corrected for best focus. Alignment is the position of the eyes relative to the target. Stereoacuity is the perception of depth. Visual processing is a higher level of visual interpretation.

### Sensory Domain Assessments Recommended

The link for the NIH Toolbox for the sensory domain assessments is: https://www.healthmeasures.net/explore-measurement-systems/nih-toolbox/intro-to-nih-toolbox/sensation

#### Near Visual Acuity

A paradigm and software for the Near Visual Acuity Test for Infants and Young Children was developed and an advanced prototype was readied for reliability and validity testing.

#### Ocular Alignment

A paradigm and software for a test of ocular alignment was developed and an initial prototype was readied for reliability and validity testing.

#### Questionnaire for Dizziness, Eye, and Balance Function (Q-DEB)

A questionnaires for each of three age groups was developed. Limited feasibility testing was achieved but further work was needed.

#### NIH Toolbox Dynamic Visual Acuity Test

Modification for this test to be performed on the iPad was approved, but this required replacement of the gyro or modification of it to enable communication with an iPad. Due to difficulties with the manufacturer, this was not successful. Use of the camera within the iPad was attempted. The test requires that the head is rotated right to left, slightly, and must achieve a rate of 180° per second. Once this rate is achieved, the optotype (letter) to be identified is displayed. This places a tremendous load on the processor, which is not easily achieved on the iPad. Currently, a new approach using the gyro built in to the iPod Touch is being investigated.

#### NIH Toolbox Balance Test

Similar to the Dynamic Visual Acuity Test, conversion of this test for use on an iPad required alternative software and hardware. The team was successful in the use of an iPod Touch for monitoring and recording the excursion and rate of sway in standing. Preliminary data indicated that the measures do change under the various test conditions, as they should. However, due to the use of a new device, reliability and concurrent validity to the original test is desirable. Further work is required.

#### Hearing Acuity

Preliminary work to develop software specifications for this measure took place. Additional work is need for reliability and validity testing.

### Sensory Domain Assessment Schedule

In Table 2, the recommended sensory domain assessments are listed along with the age recommended for their assessments. These ages were selected based on the ages of assessment to be included in the National Children's Study.

**Table 2:** Recommended sensory assessments.

**Domain**	**Construct**	**Measure**	**Birth**	**8 m**	**14 m**	**21 m**	**3 y**	**4 y**	**5 y**	**7 y**	**9 y**	**11 y**	**13 y**	**15 y**	**17 y**	**19 y**	**21 y**
Sensory	Vision	Near acuity	X	X		X											
Sensory	Vision	Distance acuity: at 10 ft (3 m)					X		X	X			X				X
Sensory	Vision	Alignment: using automated method		X		X	X		X	X			X				X
Sensory	Vision	Stereoacuity					X		X	X			X				X
Sensory	Vision	Refraction: using automated method		X		X	X		X	X			X				X
Sensory	Vision	Processing (reading)								X							X
Sensory	Hearing	Hearing sensitivity: newborn screening															
Sensory	Hearing	Tympanometry and acoustinc reflex		X	X	X	X										
Sensory	Hearing	Question regarding hearing			X	X											X
Sensory	Hearing	Behavioral hearing threshold—acuity					X			X		X				X	
Sensory	Hearing	Auditory processing: words in noise								X		X				X	
Sensory	Vestibular	Vestibular Ocular Reflex (VOR) function															
Sensory	Vestibular	VOR to slow head movement		X	X												
Sensory	Vestibular	Nystagmus—resting		X	X												
Sensory	Vestibular	Vertigo or dizziness or sense of movement (questionnaire)			X				X		X						
Sensory	Vestibular	Dynamic visual acuity						X			X			X			
Sensory	Vestibular	Vestibulospinal function															
Sensory	Vestibular	Labyrinthine righting: reflex: head control		X													
Sensory	Vestibular	Labyrinthine righting, sitting: supported, unsupported		X	X												
Sensory	Vestibular	Body control: Landau reaction—suspend prone		X													
Sensory	Vestibular	Postural control: Tilt reactions sitting (vision occluded/destabilized)			X	X											
Sensory	Vestibular	Postural control: standing—assume, hold indep, first steps, walk			X												
Sensory	Vestibular	Postural control: modified GHENT				X	X										
Sensory	Vestibular	Postural control: NIH Toolbox BAM (balance EO EC on floor and foam)						X	X		X			X			
Sensory	Somatosensory detection	Detection: light touch—monofilament at 11 gm pressure			X	X					X						
Sensory	Somatosensory detection	Detection: pain/pin prick (later sharp dull) APGAR															
Sensory	Somatosensory detection	Detection: joint position sense						X						X			
Sensory	Somatosensory detection	Detection: temperature: cold					X						X				
Sensory	Somatosensory avoidance	Avoidance: over-sensitivity (Inf Sensory Profile Q)			X	X	X										
Sensory	Somatosensory threshold	Threshold: light touch (hand, foot) monofilament test								X			X				X
Sensory	Somatosensory threshold	Vibration—graduated tuning fork							X		X					X	
Sensory	Somatosensory discrimination	Sharp/dull (dropped)															
Sensory	Somatosensory discrimination	Stereognosis: identify known objects (5) by touch alone					X		X				X			X	
Sensory	Somatosensory sensitivity	Sensitivity: (detection) neonate acceptance/rejection															
Sensory	Taste	Regional and whole mouth taste sensitivity:								X						X	
Sensory	Taste	Taste preference: taste-odor reactivity questionnaire															
Sensory	Taste	Pregnancy food frequency questionnaire															
Sensory	Taste	Sucrose preference test						X			X						
Sensory	Olfaction	Odor detection orient to maternal odor															
Sensory	Olfaction	Odor identification					X				X			X			X
Sensory	Olfaction	Odor identification and hedonic response									X			X			X
Sensory	Olfaction	Flavor identification: jellybean test							X								

### Sensory Assessments in Need of Development

Despite the great strides made in identification of important measures of sensory system health, there is work that should be completed to yield valid and reliable tools for use by researchers and clinicians. Specifically, development of the approved measures should be completed. For each of the measures approved for development or accepted for inclusion in the NCS, a brief description of ‘recommended next steps' is provided below. See Final Reports for the four measures for which at least partial development was completed for more detailed information. In addition, a brief discussion is provided at the end of this section regarding the lack of measures of somatosensation.

#### Vision

The test for Near Visual Acuity for Infants and Young Children, based on the grating visual acuity paradigm, was developed (software) for administration with an iPad. This test is now ready for feasibility testing (resulting in possible software updates), followed by reliability and validity testing. This would be the first available inexpensive, easy to administer test for this construct, and further development should be pursued.

The Ocular Alignment measure requires use of the iPad camera and measurement of the alignment of both eyes relative to a target. Software work on an initial prototype was completed. Measurement of ocular alignment is precise and even small shifts of the subject in relation to the camera have the potential to create measurement errors. The team has utilized an iterative development process to mitigate the noise caused by movement or glares/shadows in the data used for the calculation of results; however, reliability of the application continues to be a challenge. Additional cycles of development and feedback from vision experts could yield solutions that would allow for greater flexibility with testing conditions. The user interface of the current application is designed to maximize flexibility for continued design and testing.

#### Hearing

Preliminary work was completed on identifying the parameters and technical requirements of the Hearing Sensitivity Test, though software development was not completed. Software development should be completed, with modules that enable testing from 3 through 20 years of age. The test would then be ready to establish reliability and validity. Similar to the visual acuity measure, this would be the first readily available, inexpensive, easy to administer test of this construct for young children. No test for hearing acuity was published for the NIH Toolbox.

#### Vestibular

Within this sensory construct, there are three measures of concern: (1) the questionnaire (Q-DEB), (2) conversion of the NIH Toolbox Dynamic Visual Acuity Test (DVA) and (3) NIH Toolbox Standing Balance Measure for use with an iPad. It is important to note that measuring or testing of vestibular system health is currently limited to expert clinicians and researchers in the field, and requires equipment costing over $100,000. The DVA and Standing Balance measures are a good first step in accessibility of an easy-to-use, inexpensive, valid and reliable test of the VOR and VSR. However, work is needed for Dynamic Visual Acuity to be available and ready for use on an iPad, and some additional concurrent validation data for Standing Balance would be helpful.

For the Q-DEB, questionnaires were developed for use with children 1 through 20 years of age. Three age group versions were created: (1) 1–5 years of age, (2) 6–12 years, and (3) 13–20 years of age. For the two older groups, there is a parent report/questionnaire and a child/self-report questionnaire. Preliminary feasibility was completed, as was some validation. Results are promising and demonstrate that the tool is appropriately “measuring” vestibular system health. However, to enable full use and appropriate interpretation of scoring, it is necessary to complete feasibility, and establish reliability and validity (see full measure development report for this measure). Forms should be placed in formal technology for ease of administration and scoring. This test, once fully implemented, will be easy to administer and score and will yield good information regarding vestibular health. Once reliability and validity are established, the test will be useful to researchers interested in vestibular health and the impact of environment or exposures. In addition, with full validation, the questionnaires will be of importance to clinical researchers and clinicians, and will identify children for whom diagnostic testing or follow-up is warranted.

The NIH Toolbox Standing Balance Test was modified to allow use of an iPad to administer the test. To quantify vestibular system effectiveness in balance ability, it is necessary to calculate ratio scores, particularly a ratio of condition #4 (standing on dense foam with eyes closed) relative to condition #1 (standing on solid surface with eyes open). Instead of a custom-built accelerometer, the newly adapted device uses an iPod to quantify sway. The hardware and software modifications were made, but because this is a new tool, validity to the original test (which was validated to the gold standard measure) is needed. This is particularly important to determine if previously collected normative data can be used. Due to the termination of the NCS, this could not be completed. As above, this is the only valid and reliable, low-cost test that can be completed without extensive training or expertise, and can be used for individuals aged 4 through 80 years of age. Data collection should be completed to confirm validity and to determine if use of current normative data is possible.

Work was completed in an attempt to modify the NIH Toolbox Dynamic Visual Acuity Test for use with an iPad. This test required identification of alternative hardware that communicated with an iPad and extensive processing. More work is needed to make this tool ready for large-scale use. Similar to the balance test, this is the only valid and reliable, low-cost test that can be completed without extensive training or expertise, and can be used for individuals aged 4 through 80 years of age. It is therefore recommended that continued development be pursued.

#### Pursue Test Development

As noted above, three tests of Taste and Olfaction are in need of development. The measures of interest are: (1) the Taste-Odor Reactivity and Preference Questionnaire; (2) the Pregnancy Food Frequency Questionnaire; and, (3) Flavor Identification Test. These assessments would enable measurement in infants and young children, for whom currently there are no easy-to-administer, low-cost tests. The importance of these constructs in identifying the child's ready acceptance of food, and its association with maternal diet during pregnancy is well-established.

#### Somatosensation Assessment

The somatosensory system includes peripheral receptors and central processing that enable sensing and interpretation of multiple modalities to include touch, temperature, proprioception, and nociception (106). This system enables detection and interpretation of environmental and internal stimuli. Measurement of somatosensation is important due to its lifespan contribution to motor skills, balance, walking and protection of the skin, muscles and joints during movement. Because this system guides exploration of the environment by the infant/young child, its integrity is critical to typical development of cognition and motor abilities. Furthermore, somatosensation influences non-tactile impressions, decisions and social interactions (53).

Aberrant somatosensory function has been identified in children with the following conditions/diseases: type 1 and 2 diabetes (107), autism (108), cerebral palsy (109), stroke (110, 111), head trauma (112), drug exposure *in utero*, and prematurity (113). The prevalence of each disease, according to the CDC. Although when considered independently the incidence for each disease is low, when considered as a group, the evidence indicates a deleterious effect for a large number of children in the population. Approximately one million children between birth and 5 years of age (4.2% of birth to 5 year olds in the U.S. population) have somatosensory impairments. The numbers increase when one considers the incidence of concussion and diabetes in young children (potentially adding 1.1%). Furthermore, based on the incidence of late preterm births, developmental delay and cerebral palsy, and the incidence of somatosensory problems in these children, the numbers with somatosensory impairment increases each year by ~180,000 (new births).

The major role for somatosensation in typical development and health status in young children supports the importance of its measurement in studies interested in the health of children and the effects of exposures on it. Currently, tests of these constructs that are not invasive or that do not require expertise and/or specialized equipment are not available. To address this, the team recommended development of three tests in the sub-constructs of detection, processing and avoidance: (1) light touch (detection), (2) stereognosis (processing), and (3) modified Infant Sensory Profile (avoidance). It is important to note that there are no tests of somatosensation included in the NIH Toolbox. Omission of tests or measurement of somatosensation is a major oversight.

## The Physical Health and Systems Domain^3^

The Physical Health and Systems (PHS) Domain was responsible for a wide range of constructs related to child health, development and behaviors, parent health behaviors, health assessments, and exposure variables. The PHS Domain target areas included child body size, body composition and pubertal status, physiological status, child and parent health behaviors, bio- specimens, and medical records.

### Physical Health Systems Value Proposition

The proposed measures in the PHS domain reflect constructs or factors that are related to child growth and development or have direct and/or indirect influence on child health and long-term disease risk. Many factors have relationships to cardiovascular or metabolic disease risk status. The health behaviors included may be viewed as both important behavioral outcomes and key potential influences on health and disease outcomes.

### Physical Health Systems Constructs and Assessments Recommended

Five target areas were identified for assessment. These target areas included: (1) Body Size, Body Composition, and Pubertal Status; (2) Physiologic Status; (3) Health Behaviors; (4) Bio-specimens; and, (5) Medical Records. For each target area, the PHS Domain Team recommended measures based on validity, feasibility, and participant burden. Three levels of recommended measures were established. *Level 1* measures were considered the best approach for accurately assessing a particular construct. These assessments typically required substantial resources with variable participant burden. *Level 2* measures were the next best approach, typically requiring less rigorous and/or requiring fewer time points to be assessed. These assessments require fewer resources and/or burden than level 1 measures. *Level 3* measures were the least supported approach and require the fewest resources and include minimal participant burden. Level 3 assessments were considered the least rigorous of the three levels of recommended measures. Each measure proposed has been demonstrated to be reliable and valid against the accepted gold-standard or another acceptable standard for measurement of the listed construct.

For the two remaining target areas, medical records and bio-specimens, work groups were organized and preliminary discussions occurred, but there were no consensus statements for each of these two target areas for proposed variables or measurement protocols.

#### Body Size, Body Composition, and Pubertal Status

**Body Size**. Body length/height is an important indicator of child growth and maturation. Body size, especially when outside of normal ranges, is an indicator of development and potential developmental issues, which are important to understand both as independent variables and as modifiers of other health outcomes. Body length will be measured until the 24-month measurement period, or until the child can stand (whichever occurs first), at which point standing height will be measured (114, 115).

**Weight** is an important variable as it relates to growth and development, both as an indicator of nutritional disorders (underweight and overweight) and/or as a risk factor for various diseases. Weight will be measured at 6 months, again at 12 months and every year thereafter. For measurements when the child is unable to stand (6, 12 months), the adult will hold the child and the scale will be tared to the adult's weight. At 24 months and beyond, weight will be measured with the child standing independently on the scale.

**Waist Circumference** is a measure that is used to assess body fat distribution and abdominal adiposity. Abdominal adiposity is associated with several cardio-metabolic diseases, including obesity, diabetes, CVD, and CHD. Waist circumference will be evaluated at 12 months of age and annually until the age of 21 years, using an insertion measuring tape (116–118).

**Body Composition** is a critical anthropomorphic characteristic that is associated with multiple developmental phenomena and health outcomes. Body composition (percent fat) will be estimated from Bioelectrical Impedance Analysis (BIA). Bioelectrical Impedance Analysis (BIA; primary measure of body composition) is proposed as the measure of body composition beginning at 48 months. BIA is a non-invasive technique that measures the body tissue impedance to an electrical current, which can then be used to determine the composition of fat and fat-free mass in the body (119, 120).

**Sexual Maturity Assessment** by sex characteristics provides information about a child's maturation status relative to physiologic age rather than chronological age (121). It is an important outcome, but also an important modifier and/or moderator of several health outcomes, behaviors, and risk factors. Sexual maturity will be assessed based on secondary sex characteristics, and age at menarche in females. Assessments will be made annually beginning at age 8 years and continuing to 16 years or until Tanner stage 5 is reached (adult or mature state) (122).

#### Physiologic Status

**Blood Pressure** is a vital sign, and elevated blood pressure in children is a risk factor for future development of hypertension and other cardiovascular diseases. Among children and adolescents, hypertension is positively associated with several cardiovascular risk factors, including glucose, adiposity, fibrinogen, cholesterol, fibrinogen and triglyceride levels. Because of its potential value in predicting future health outcomes and because hypertension is itself an important health outcome, blood pressure should be measured at regular intervals as children develop (123, 124).

**Lung Function** refers to the effectiveness with which air can be moved in and out of the respiratory system. It is typically measured *via* spirometry, although other methods exist. Assessment of lung function is critical to understanding child health as it reflects an essential physiologic function, and as an indication of disease status. The Vitalograph and other portable spirometers have good reproducibility and agreement with previously-established pneumotachograph flowmeters (125, 126).

**Physical Fitness** will be assessed using cardiorespiratory fitness (CRF) which is the ability of the circulatory, respiratory, and muscular systems to support oxygen during whole body physical activity. Evidence indicates that youth CRF is associated with adiposity in adulthood, waist circumference, glucose levels, insulin levels, HDL-C and adiposity, all of which are risk factors for several non-communicable diseases, including cardiovascular disease, coronary heart disease, type 2 diabetes mellitus and stroke (127). The Sub-Maximal Treadmill test with NHANES protocol is recommended to assess cardiorespiratory fitness.

#### Health Behaviors

##### Health Behavior (Child)

***Sleep*** *(child)*. Sleep is a complex physiological process that is important for the health and well-being of children. In particular, disturbed or inadequate sleep duration is associated with increased risk of obesity (128), decreased cognitive functioning (129), difficulty regulating emotions (130), and several additional negative outcomes. During childhood and adolescence, abnormal sleep behavior is a risk factor for development of overweight and obesity and may have other negative developmental and health consequences. It is recommended that sleep routine questions be adapted from the Avon Longitudinal Study of Parents and Children (ALSPAC), My Young Baby Girl Questionnaire and the Brief Infant Sleep Questionnaire (BISQ).

The child sleep environment will be assessed with items from the State and Local Area Integrated Telephone Survey, National Survey of Early Childhood Health, Pregnancy Risk Assessment Monitoring System (PRAMS), ALSPAC and the Pediatric Sleep Questionnaire (PSQ). Modifications to the questionnaires are recommended (131).

***Physical Activity (child)***. Higher physical activity levels provide physical and psychological benefits during childhood and adolescence. Specifically, physical activity in children can help to control body composition and weight, build lean muscle, promote strong bone and joint development, improve cardiovascular and metabolic health markers, reduce symptoms of depression and decrease the risk of obesity (132). Physical activity is an important health outcome and also is a moderator of several risk factors and disease states. It is recommended that physical activity be measured with a physical activity monitor (accelerometer) which is a lightweight device that is worn on the non-dominant wrist and/or on the right hip. This device provides an objective measure of physical activity. In addition, it is recommended that the NHANES Physical Activity Questionnaire be administered.

***Diet (child)***. Feeding and dietary intake are associated with growth and development, immunity, development of weight status, cardiometabolic risk profile, and development of disease states. Dietary intake also can provide information about exposure to certain food toxins or allergens that are important in health status. The Infant Feeding Questionnaire is recommended for infants 0–12 months (133, 134). For those from 2 years and older, the ASA24, a web-based, automated self-administered 24-h recall assessment is recommended.

***Risk Behaviors (child)***. Child participation in risk behaviors, such as jumping from high places or riding a bicycle without a helmet, is associated with injury outcomes and may predict future participation in risky behaviors. Risk behaviors that may affect child and adolescent safety include behaviors contributing to unintentional injury and violence, such as seat belts use, wearing a bike helmet, proper use of a car safety seat, exposure to guns/weapons, bullying, and driving practices. The Injury Behavior Checklist is recommended for assessing a child's participation that could lead to injury (135, 136).

***Time Use (child)***. Assessment of time use is a way to understand behavior prioritization as a child becomes more independent. Time use diaries or questionnaires can provide information about how much time is spent in certain activities and the social and physical environments associated with those behaviors. Understanding time use is relevant to a child's health in that it provides documentation of health and risk behaviors in which a child participates and can provide information about exposures to various environments. Time use will be assessed *via* child self- report beginning at age 9 and continuing annually through age 21.

***Media Use (child)***. Media use and screen time are important factors in determining time spent in sedentary behavior and may also provide information about educational/learning resources and time and other exposures. Viewing of Media and Media Exposure Surveys from the NCS Vanguard Study are recommended for assessment.

##### Health Behaviors (Parent)

***Sleep (parent)***. Parental sleeping habits can have several direct and indirect influences on child health. Poor infant sleep has been associated with poor maternal sleep, which in turn is associated with increased postnatal depression (137). Parental sleep patterns can directly and indirectly affect child sleep and also impact caregiving, depression and anxiety of the parent. Assessing Child and Maternal Sleep in the Early Years Survey used in the NCS Vanguard Study are recommended.

***Alcohol, Tobacco, and Substance Use (parent)***. Parental substance use has direct and indirect effects on child health outcomes. Infants who are exposed to substances during gestation are born at younger gestational age and are at significant risk of low birth weight (138). Substance use clearly can impact child health *in utero*, as an environmental exposure, and as a factor in the home environment, caregiving, modeling, and child participation in risk behaviors. Alcohol, tobacco, and substance use will be assessed of the biological mother or primary caregiver as well as the secondary caregiver in the home (if present). Survey items that have been compiled from the National Survey on Drug Use and Health (modified), Alcohol Disorder and Associated Disabilities Interview Schedule (4th edition), National Epidemiologic Survey on Alcohol and Related Conditions, National Health Interview Survey, NHANES. These survey items were used in the NCS Vanguard study and are recommended.

***Physical Activity (parent)***. Regular physical activity is essential to experience health benefits, such as decreased risk for coronary heart disease, stroke, type 2 diabetes, osteoporosis, and depression, and improved physical fitness and aerobic capacity (6, 7). Parent, and specifically biological mother, physical activity can play an important role in a child's fetal growth and development, as well as development of fatness and participation in physical activity behaviors. The Long-Form International Physical Activity Questionnaire (IPAQ) is recommended first and the Short- Form of IPAQ is recommended second (139, 140).

***Diet (Parent)***. Dietary Intake of the parent may play a significant role in the development of many diseases and health status in children. Biological mother's diet plays a role in taste preferences of the child, and parental eating behaviors can influence child eating preferences and patterns and the development of fatness. Parents and caregivers are role models who create the home food environment and control the types of foods which are available and accessible to children at home. The ASA24 web-based, automated, self-administered 24-h recall is the recommended assessment.

***Parents Behaviors to Reduce Risk***. Safety behaviors practiced by parents can help reduce risk for unintentional injury or death in children. Use of prevention equipment like smoke alarms, seat belts and child safety seats are all parental practices that can protect children. Firearm safety and security is also an important factor that can contribute to child safety and opportunity for avoidable risk. Items from the Risk and Safety Behaviors Survey were selected and used in the NCS Vanguard study.

#### Biospecimens

Biospecimens are required for assessment of a large and diverse set of variables that relate to multiple health constructs. The recommendations in this section organized by construct, with biospecimen sample options listed in the recommended measures section. It is recommended that biospecimens be collected from the child, biological mother, biological father, and primary caregiver (if not either biological parent) at specified intervals through the child's life-course. In general, collection of biospecimens at regular intervals is more important than the collection of the samples at any specific age points. Efforts should be made to align the child and parent/caregiver sample collection for ease of sample collection. Additionally, tiered approaches can be offered such that if a child (or adult) does not want to give a blood sample, a saliva and urine sample would be collected.

##### Cardio-Metabolic Health Biomarkers

Cardio-metabolic health biomarkers are important indicators of both health and disease. These biomarkers include, but are not limited to: blood lipids (cholesterol, triglyceride), inflammatory markers, glucose, insulin, apolipoproteins, nitric oxide, leptin, fibrinogen, fatty acids, electrolytes, troponin, creatine kinase, kidney function, oxidative stress, homocysteine, and adipokines. Recommend measures: blood, saliva, and urine.

##### Immune Status/Inflammatory Response

Stress, physiologic injury and immune function can all be related to disease status, either in the precursor or the consequences pathway. Biomarkers related to immune status and/or inflammatory processes are important independent outcome variables as well as indicators of disease process or status. Immune status biomarkers include, but are not limited to: white blood cell count, lymphocytes, leukocytes, cytokines, immunoglobulin A, nitric oxide, cytokines, and interleukin. Recommend measures: blood and saliva.

##### Exposure to Drugs/Toxins

Biomarkers of exposure to drugs/toxins include, but are not limited to: fatty-acid ethyl esters for alcohol exposure (hair), and apolipoprotein-E (drug-induced liver injury). Recommended Measures: Hair, Blood, and Saliva.

##### Exposure to Environmental Contaminants

Biomarkers of exposure to environmental contaminants include, but are not limited to: 1-hydroxypyrene (urine), DNA adducts, heavy metals in serum and/or urine, and oxidative stress markers. Recommended Measures**:** Blood, Saliva, Urine, Hair, and Nail.

##### Genetic Profile

The genetic profile markers include DNA and mRNA. Recommended Measures: Blood.

##### Birth Factors (Child Only)

Biomarkers of birth factors include, but are not limited to: Meconium fatty acid ethyl ester (fetal alcohol exposure), pro-inflammatory cytokines. Recommended Measures: Blood, Placenta, Cord Blood, and Meconium.

##### Pregnancy Profile/Health (Bio-Mom Only)

Recommended Measures: Blood, Saliva, Urine, Breast Milk, Placenta, and Breast Milk.

##### Hematologic Status

Biomarkers of hematologic status include, but are not limited to: red blood cells, white blood cells, platelets, hemoglobin, hematocrit, mean corpuscular volume, blood glucose, calcium, electrolytes, iron status (folate and ferritin), and blood coagulation.

Recommended Measures: Blood.

##### Endocrine Function

The endocrine system of the body consists of several glands that secrete hormones that can have many target organs and/or modes of action. The endocrine system plays a role in metabolism, growth and development, tissue function, sleep, digestion, respiration, excretion, mood, stress, lactation, movement, reproduction, and sensory perception. Biomarkers of endocrine function include, but are not limited to: Chromogranin-A, calcium, free thyroxine, thyroid stimulating hormone, follicle stimulating hormone, thyroid hormones, serum cortisol, sex hormones, glycosuria, proteinuria, insulin, and growth hormone. Recommended Measures: Blood and Urine.

##### Liver Function

Biomarkers of liver function include, but are not limited to: aminotransaminases, bilirubin, bile acids, protein levels/amino acids, cytokines, apolipoprotein-E, steroids, and platelet count. Recommended Measures: Blood and Urine.

#### Medical Records

##### Medical Records (Child)

Several components of medical history and current health status are informative in determining a child's current health status, predisposition and/or risk for future disease. It is important to understand medical conditions and events as they happen in the child's life and as such, several components of medical history will be assessed regularly as part of the NCS. Information on the child, including utilization of care, diagnoses, medication/prescription, immunizations, and the birth/labor and delivery record will be obtained.

##### Medical Information (Parent/Adult Caregiver)

Parental medical history is important to obtain given genetic predisposition for disease and health risks associated with parental medical conditions. Information on the biological mother's prenatal experience including labs, physical exam, ultrasound, blood type, and prenatal care will be obtained at birth. Information on utilization of care, diagnoses, and medication/prescription use will be obtained from the biological mother, biological father, and/or primary caregiver (if not either biological parent) at birth (except primary caregiver), 4, 9, and 16 years.

### Physical Health and System Assessment Schedule

The PHS Domain provided recommendations for measures of relevant constructs in three categories: (1) body size, body composition, and pubertal status, (2) physiologic status, and (3) health behaviors. Table 3 includes a timeline for the measurements developed.

**Table 3:** Physical health assessments.

**Domain**	**Construct**	**Measure**	**Birth**	**8 m**	**14 m**	**21 m**	**3 y**	**4 y**	**5 y**	**7 y**	**9 y**	**11 y**	**13 y**	**15 y**	**17 y**	**19 y**	**21 y**
Physical	Body size and composition	Length	X	x	x	x											
Physical	Body size and composition	Height					x	x	x	x	x	x	x	x	x	x	x
Physical	Body size and composition	Weight	X	x	x	x	x	x	x	x	x	x	x	x	x	x	x
Physical	Body size and composition	Waist circumference	X	x	x	x	x	x	x	x	x	x	x	x	x	x	x
Physical	Body size and composition	BIA					x	x	x	x	x	x	x	x	x	x	x
Physical	Body size and composition	Sexual maturation								x	x	x	x	x			
Physical	Physiology	Blood pressure (and HR)			x	x	x		x	x	x	x	x	x	x	x	x
Physical	Physiology	Pulmonary function							x	x	x	x	x	x	x	x	x
Physical	Physiology	Cardio-respiratory fitness								x	x		x	x	x		
Physical	Health behaviors	Sleep		x	x	x	x	x	x	x	x	x	x	x	x	x	x
Physical	Health behaviors	Physical activity behavior (quest)			x	x	x		x	x	x	x	x	x	x	x	x
Physical	Health behaviors	Physical activity (accelerometer)						x	x	x	x	x	x	x	x	x	x
Physical	Health behaviors	Infant feeding		x	x												
Physical	Health behaviors	Dietary intake				x	x	x	x	x	x	x	x	x	x	x	x
Physical	Health behaviors	Child risk behaviors (unintentional injury)			x	x	x	x	x	x	x	x	x	x	x	x	x
Physical	Health behaviors	Time use									x						
Physical	Health behaviors	Media use		x	x	x	x	x	x	x	x	x	x	x	x	x	x
Physical	Medical records	Utilization of care			x			x			x			x			x
Physical	Medical records	Diagnoses			x			x			x			x			x
Physical	Medical records	Medication/prescriptions			x			x			x			x			x
Physical	Medical records	Immunizations			x			x			x			x			x
Physical	Medical records	Birth record/L&D record		x													

### Physical Health System Assessments Under Development

The PHS Domain team proposed two development studies for measures that were potential measurement options for body composition and cardio-respiratory fitness. A calibration study of Bio-electrical Impedance (BIA) is needed due to limited information about use of BIA in young children and lack of estimation equations for body composition in diverse samples. A validation of non-exercise models for prediction of cardiorespiratory fitness (CRF) in children was proposed as a more feasible method of estimating fitness in the large studies. Non-exercise models of CRF are valid in adults but no non-exercise models exist for children. Using existing, large datasets, we proposed to investigate the feasibility and validity of non-exercise models of estimating CRF in children.

## In Summary

This paper documents the work of three scientific teams focusing on the motor, sensory, and physical health and system domains that were charged to make recommendations to the National Children's Study regarding assessment of children from birth to 21 years in these three domains. Domain constructs were identified and measures of these constructs were proposed along with recommended ages for assessment. The committees also identified assessment instruments that were in need of revision or new instruments developed.

## Author's Note

The three chairs are listed in alphabetical order. Clark chaired the Motor Domain team; Pate chaired the Physical Health and Systems Domain team; and, Rine chaired the Sensory Domain team. After the three chairs, the remaining committee members are listed in alphabetical order. At the start of each domain section, the members of that domain committee are listed in a footnote.

## Data Availability Statement

The original contributions presented in the study are included in the article/supplementary material, further inquiries can be directed to the corresponding author/s.

## Author Contributions

JC chaired the Motor Domain team. RP chaired the Physical Health and Systems Domain team. RR chaired the Sensory Domain team. JC, PD, DD, SD, JH, PK, SM, RM, KM, KN, TS, DS, ET, and SH contributed equally to the domain reports. All authors contributed to the article and approved the submitted version.

## Funding

This paper was supported by the NIH National Children's Study (NCS).

## Conflict of Interest

The authors declare that the research was conducted in the absence of any commercial or financial relationships that could be construed as a potential conflict of interest.

## Publisher's Note

All claims expressed in this article are solely those of the authors and do not necessarily represent those of their affiliated organizations, or those of the publisher, the editors and the reviewers. Any product that may be evaluated in this article, or claim that may be made by its manufacturer, is not guaranteed or endorsed by the publisher.

## Footnotes

^1^Team members: JE Clark, chair; D Damiano; S. Magasi; K. Newell; T. Sanger; D. Sugden.

^2^RM Rine, Chair; JM Holmes; R. McCreery; J. Christy; P. Dalton.

^3^R Pate, Chair; K McIver; E Taveras; S Daniels; PT Katzmarzyk.
